# *Rhodiola rosea* as an adaptogen to enhance exercise performance: a review of the literature

**DOI:** 10.1017/S0007114523001988

**Published:** 2024-02-14

**Authors:** Grant M. Tinsley, Andrew R. Jagim, Gregory D. M. Potter, Dan Garner, Andrew J. Galpin

**Affiliations:** 1 Department of Kinesiology and Sport Management, Texas Tech University, Lubbock, TX 79409, USA; 2 Sports Medicine, Mayo Clinic Health System, La Crosse, WI, USA; 3 Greg Potter PhD Limited, Brighton, East Sussex, UK; 4 BioMolecular Athlete, LLC, Wilmington, DE, USA; 5 Center for Sport Performance, Department of Kinesiology, California State University, Fullerton, CA, USA

**Keywords:** Arctic root, Dietary supplement, Golden root, Physical performance, Rosavin, Roseroot, Salidroside

## Abstract

*Rhodiola rosea* (RR) is a plant whose bioactive components may function as adaptogens, thereby increasing resistance to stress and improving overall resilience. Some of these effects may influence exercise performance and adaptations. Based on studies of rodents, potential mechanisms for the ergogenic effects of RR include modulation of energy substrate stores and use, reductions in fatigue and muscle damage and altered antioxidant activity. At least sixteen investigations in humans have explored the potential ergogenicity of RR. These studies indicate acute RR supplementation (∼200 mg RR containing ∼1 % salidroside and ∼3 % rosavin, provided 60 min before exercise) may prolong time-to-exhaustion and improve time trial performance in recreationally active males and females, with limited documented benefits of chronic supplementation. Recent trials providing higher doses (∼1500 to 2400 mg RR/d for 4–30 d) have demonstrated ergogenic effects during sprints on bicycle ergometers and resistance training in trained and untrained adults. The effects of RR on muscle damage, inflammation, energy system modulation, antioxidant activity and perceived exertion are presently equivocal. Collectively, it appears that adequately dosed RR enhances dimensions of exercise performance and related outcomes for select tasks. However, the current literature does not unanimously show that RR is ergogenic. Variability in supplementation dose and duration, concentration of bioactive compounds, participant characteristics, exercise tests and statistical considerations may help explain these disparate findings. Future research should build on the longstanding use of RR and contemporary clinical trials to establish the conditions in which supplementation facilitates exercise performance and adaptations.

## Graphical abstract

**Figure:**
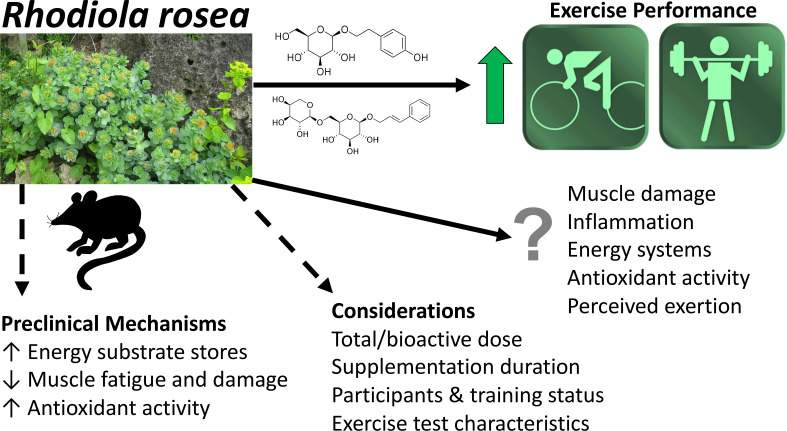

*Rhodiola rosea* (*RR*) is a flowering perennial plant found in Arctic regions of Europe, Asia and North America. Known by several other names – including roseroot, rosenroot, golden root, arctic root and more – this member of the Crassulaceae family has been used for medicinal purposes for centuries, with the Greek physician Dioscorides describing medicinal application in 77 AD^(1,2)^. The physiological effects of *RR* ingestion are thought to arise from its roles as an adaptogen, a term historically applied to substances that cause ‘a state of non-specifically increased resistance to stress’^(3)^. Adaptogens can be defined as substances that promote physiological resilience, resistance to stress and maintenance or restoration of physiological function when homoeostasis is challenged. In this regard, adaptogens may enhance physical and cognitive performance under duress, as well as general well-being, and several purported adaptogens are experiencing increased popularity in the dietary supplement industry^(4)^. The adaptogenic actions of *RR* are primarily attributed to bioactive compounds within the root, with salidroside and rosavin often noted as the most influential compounds^(5)^. As a result, many commercial *RR* preparations are standardised to specific concentrations of salidroside and rosavin. However, at least 109 chemical compounds have been identified in *RR* ^(2)^. Collectively, these bioactive components have been observed to exert anti-stress and anti-fatigue effects, as well as enhance aspects of cognitive and physical performance, in part through their antioxidant properties^(6,7,8,9)^. The effects of *RR* on cognitive and physical function could also relate to interactions with components of physiological stress-response systems, such as monoamine neurotransmitters (e.g. serotonin and catecholamines) and opioid peptides (e.g. *β*-endorphins)^(10)^.

The longstanding medicinal use of *RR* and the marked popularity of dietary supplements containing extracts of this plant^(4)^ warrant cohesive summaries of research detailing its physiological effects. While *RR* has been widely studied for aiding mental health and cognitive function^(11)^, as well as general stress and fatigue resistance^(10)^, the purpose of this narrative review is to describe the potential roles of *RR* as an adaptogen within the context of physical performance. We begin by considering preclinical research on *RR*’s putative mechanisms of action and then summarise the available clinical research on the efficacy of *RR* supplementation.

## Potential mechanisms of physical performance benefits

Multiple mechanisms have been proposed to explain the potential ergogenic effects of *RR* on exercise performance, recovery and long-term adaptations to exercise training. Several rodent studies that have demonstrated improvements in exercise performance following supplementation have probed the means by which *RR* acts^(12,13)^. Four weeks of *RR* ingestion (5–125 mg/d) increased resting liver glycogen content and attenuated muscle glycogen depletion during 90-min unloaded swimming exercise in Wistar rats, although the mechanism for these findings was not established^(12)^. *RR* prolonged time-to-exhaustion (TTE) during weight-loaded swimming by 21–65 %, with increasing doses providing greater benefits. Compared with the control group, *RR* supplementation reduced post-exercise fatigue biomarkers, including glutamic oxaloacetic transaminase, glutamic pyruvic transaminase and lactate dehydrogenase, and supplementation increased skeletal muscle and liver tissue oxygenation and expression of proteins involved in TAG synthesis (sterol regulatory element-binding protein-1 and fatty acid synthase)^(12)^. Other work has corroborated the reduction in post-exercise lactate dehydrogenase following 30 d of *RR* supplementation and also finding reductions in creatine kinase, suggesting an attenuation of exercise-induced muscle damage during strenuous activity^(14)^. A separate investigation reported that 6 d of supplementation with 50 mg/kg/d of *RR* (3 % rosavin, 0·9 % salidroside) administered 30 min prior to daily exercise sessions increased swimming TTE by 25 % in Sprague-Dawley rats^(13)^. Mitochondrial ATP content, as estimated by a bioluminescence assay quantifying ATP reactivity with recombinant firefly luciferase^(15)^, was better preserved following exercise in the supplemented group, implying *RR* may improve mitochondrial ATP synthesis during or after intense exercise. In rat skeletal muscle cells, isolated salidroside has been found to activate AMP-activated protein kinase^(16)^, a master regulator of exercise signalling pathways that senses cellular energy status and exerts numerous downstream effects on carbohydrate and lipid metabolism at times of energy stress^(17)^. Together, these studies show that *RR* supplementation may enhance endurance exercise performance in rodents by countering fatigue associated with changes in cellular bioenergetics.

Additional research has assessed the potential roles of *RR* as an antioxidant, which may be relevant to exercise training, recovery and adaptation. While reactive oxygen species have important signalling roles that affect physiological adaptations to exercise, the antioxidant status of individuals influences whether antioxidant supplementation will have positive, negative or null effects on exercise performance and adaptations^(18)^. As such, potential antioxidant effects of bioactive compounds should be considered alongside baseline antioxidant status, which is influenced by dietary intake, adaptations to exercise and numerous other factors. In a rodent model, Huang *et al*.^(19)^ demonstrated the free radical-scavenging activity of several of *RR*’s bioactive phytochemicals (e.g. salidroside, rosavin, rosin and rosarin) and found *RR* supplementation enhanced weight-loaded swimming performance. In this study, 4 weeks of *RR* supplementation increased liver expression of the antioxidant enzymes catalase, manganese superoxide dismutase and copper/zinc superoxide dismutase; suppressed exercise-induced increases in oxygen-free radicals in blood, liver and skeletal muscle and reduced levels of the lipid peroxidation product malondialdehyde^(19)^. Other work has highlighted the potential of isolated salidroside to increase antioxidant enzyme activity, bolster liver glycogen and improve exercise performance following 4 weeks of supplementation^(20)^. Collectively, murine research that has assessed antioxidant activity alongside exercise performance has generally reported that ergogenic effects of *RR* supplementation are concurrent with augmented antioxidant defence systems. In these studies, *RR*’s antioxidant activity appears not to hinder beneficial exercise adaptations due to suppression of signalling by reactive species, as has been reported for certain other antioxidant supplements, such as high doses of vitamins C and E (1000 mg/d vitamin C and 400 mg/d vitamin E)^(21)^. It is also possible that the other advantageous effects of supplementation may outweigh any detrimental effects resulting from acutely increased antioxidant activity. Additionally, it is plausible that the antioxidant status of the animals used in this research may have been conducive to demonstrating ergogenic effects of a supplement with antioxidant activity^(18)^.

Compared with endurance-based exercise models, there has been less research on the potential mechanisms by which *RR* modifies responses to resistance training. Roumanille *et al*.^(22)^ studied both acute and chronic effects of *RR* supplementation (2 % rosavin, 1 % salidroside) in rats performing climbing resistance exercise. The authors observed no effects of supplementation on post-exercise skeletal muscle protein synthesis. In keeping with this finding, *RR* had no influence on muscle growth, strength or power following 4 weeks of exercise training plus supplementation. Clearly, additional mechanistic research is needed to establish any influences of *RR* on adaptations to resistance training. While yet to be demonstrated in a preclinical model of resistance training, one might speculate that the aforementioned *RR*-induced reductions in exercise-induced muscle damage and improvements in cellular bioenergetics could positively affect resistance training.

In summary, preclinical data suggest that *RR* modulates energy substrate storage and use, reduces fatigue and muscle damage and increases antioxidant status. Despite the promise of this research, clinical studies are needed to identify whether these findings translate to humans, as well as to elucidate ergogenic dosing protocols.

## Studies of humans

### Study characteristics

#### Literature search

Over the past two decades, more than a dozen clinical trials have examined the effects of *RR* on exercise performance and adaptations (Table 1)^(23,24,25,26,27,28,29,30,31,32,33,34,35,36,37,38,39)^. To ensure this narrative review accurately represents the current body of scientific evidence, searches were performed using several electronic databases (PubMed®, Web of Science^TM^ and Scopus), with screening of relevant articles to identify research reporting the effects of *RR* on exercise performance and related outcomes in humans. We only considered trials providing *RR* in isolation, excluding those providing it as a component of a multi-ingredient supplement. In this process, we identified sixteen trials, primarily randomised controlled trials, published between 2000 and 2023, collectively totalling 363 total participants.

**Table 1.** Human studies of *RR* supplementation for physical performance enhancement

Reference		Participants								Study characteristics						Results	
Year	Researchers	*n* (sex)	Age (years)		BM (kg)		BMI (kg/m^2^)		Training status	Design	Acute/chronic*	Supplement duration (d)	*RR* dose (mg/d)	%Salidroside; %Rosavin	Exercise testing	Exercise performance	Other outcomes
2000	Spasov *et al*.^(23)^	40 (M)	17–19		NR		NR		NR	Randomised, double-blind, placebo-controlled parallel-arm trial	Chronic	20	100 (as SHR-5)	∼2 % ∼3 %	Bicycle ergometer (physical work capacity-170 test)	↔ Physical working capacity ↓ HR	↑ Movement accuracy and speed ↔ Tapping test ↔ Mental capacity ↑ General well-being ↓ Mental fatigue
2004	Abidov *et al*.^(24)^	36 (NR)	21–24		NR		NR		Untrained	Randomised, double-blind, placebo-controlled parallel-arm trial	Chronic	36	680	NR; 60 mg total active substances	Bicycle ergometer (incremental max test)	NR	↓CRP ↓CK
2004	De Bock *et al*.^(25)^	24 (12 M, 12 F)	M: 22 F: 20	0·3 0·3	M: 72·3 F: 59·4	2·4 1·5	NR		Active	Acute: Randomised, double-blind, placebo-controlled, crossover trial Chronic: Randomised, double-blind, placebo-controlled, parallel-arm trials	Acute & Chronic	1 to 2 (Acute) 28 (Chronic)	200 60-min pre-exercise	1 % 3 %	Bicycle ergometer (incremental max test) Isokinetic dynamometer Plate-tapping test	↑TTE (acute) ↑VO2peak ↔ Maximal isometric torque ↔ Limb movement speed	↔ Sustained attention
2007	Walker *et al*.^(26)^	12 (M)	29·9	4·5	89·6	12·1	NR		Trained (>6 months RT)	Double-blind, placebo-controlled, crossover trial	Acute	4	1500 for 3 d 1000 on day of test	NR 3 %	Incremental forearm wrist flexion (to exhaustion)	↔ TTE ↔ RPE	↔ [PCr] ↔ [ATP] ↔ [Pi] ↔ [pH]
2009	Skarpanska-Stejnborn *et al*.^(27)^	22 (M)	RR: 20·4 PL: 21·0	1·20·9	RR: 78·6 PL: 83·3	10·6 7·9	NR		Trained (members of national rowing team)	Randomised, double-blind, placebo-controlled parallel-arm trial	Chronic	28	200	NR	Rowing ergometer (2000 m max test)	↔ Power output ↔ Exercise time	↓ SOD ↔ GPx ↔ TBARS ↔ CK ↑TAC ↔ Lactate ↔ Uric acid
2010	Parisi *et al*.^(28)^	14 (M)	25	5	69·4	9·4	22·2	2·4	Athletes involved in competitive sport (8·4 ± 1·4 h training/week)	Double-blind, placebo-controlled, crossover trial	Chronic	28	170	NR	Bicycle ergometer (cycle to exhaustion at 75 % VO2max)	↔ VO2max ↔ HR ↔ RPE ↔ TTE	↓NEFA ↓lactate ↓CK ↔ for all others
2013	Noreen *et al*.^(29)^	18 (F)	22·0	3·3	56·6	6·2	NR		Recreationally active	Randomised, double-blind, placebo-controlled, crossover trial	Acute	1	∼170 (3 mg/kg) 60-min pre-exercise	1 % 3 %	Bicycle ergometer (6-mile time trial)	↓ Time to completion ↔ Average power ↔ Average cadence ↓ HR (warm-up only) ↓ RPE	↔ Lactate (blood) ↔ Cortisol (saliva) ↔ Alpha amylase (saliva) ↔ Profile of Mood States ↔ Stroop Colour Test
2014	Duncan *et al*.^(30)^	10 (M)	26·0	6	67·7	6·3	NR		Recreationally active (3–10 h physical activity/week)	Randomised, double-blind, placebo-controlled, crossover trial	Acute	1	∼203 (3 mg/kg) 60-min pre-exercise	NR	Bicycle ergometer (30-min at 70 % VO2max)	↔ EE ↔ Substrate oxidation ↔ HR ↓ RPE	↑ Arousal ↑ Pleasure ↑ Vigour
2014 2015	Shanely *et al*.^(31)^ Ahmed *et al*.^(32)^	48 (35 M, 13 F)	RR: 40·3 PL: 43·9	1·4 1·2	RR: 72·7 PL: 71·0	2·6 2·0	RR: 23·4 PL: 23·3	0·6 0·4	Trained (runners)	Randomised, double-blind, placebo-controlled parallel-arm trial	Chronic	38	600	1·4 % 3·8 %	Running (marathon race)	↔ Race performance ↔ Vertical jump ↔ DOMS	↔ Viral susceptibility ↓ Virus replication ↔ Antibacterial activity ↔ Myoglobin ↔ CK ↔ AST ↔ ALT ↔ IL-6, IL-8, IL-10 ↔ MCP-1 ↔ G-CSF ↔ CRP ↔ eHSP72
2016	Duncan *et al*.^(33)^	12 (M)	24·6	6·0	NR		NR		Recreationally active (3 to 10 h/week of recreational physical activity)	Randomised, double-blind, placebo-controlled crossover trial	Acute	1	3 mg/kg 60-min pre-exercise	1 % 3 %	Treadmill (5-km time trial run at 1 % gradient)	↔ Time to completion ↔ HR ↔ Blood pressure ↔ RPE	↔Lactate
2018	Timpmann *et al*.^(34)^	20 (M)	22·1	2·8	RR: 76·4 PL: 83·7	12·2 7·8	NR		Active	Double-blind, placebo-controlled parallel-arm trial	Chronic	8	432	2·3 % 2·7 %	Treadmill (walk to exhaustion at 6 km/h). Conducted in climate chamber (42°C, 18 % humidity).	↔TTE ↔HR ↔RPE	↔Core temperature ↔Skin temperature ↔Cortisol ↔Lactate ↔Glucose
2018	Jówko *et al.* ^(35)^	26 (M)	RR: 20·5 PL: 20·9	0·3 0·2	RR: 79·1 PL: 81·1	2·8 3·0	NR		Recreationally active	Randomised, double-blind, placebo-controlled parallel-arm trial	Chronic	28	600 for 27 d 200 additional 90-min pre-exercise	NR 3 %	Bicycle ergometer (TTE/VO2peak incremental max test)	↔ VO2peak ↔ TTE ↔ Maximum power ↑ Change in maximum power ↔ HR	↓ Reaction time ↔ Cortisol ↔ Testosterone ↔ GH ↔ CK ↑ TAC ↔ SOD ↔ Lipid hydroperoxides ↓ Lactate (at rest)
2019	Ballmann *et al*.^(36)^	11 (F)	19·4	0·8	66·2	8·5	23·3	2·5	Active (>150 min/week)	Randomised, double-blind, placebo-controlled, crossover trial	Acute	4	1500 for 3 d 500 additional 30-min pre-exercise	1 % 3 %	Bicycle ergometer (3 × 15-s Wingate anaerobic tests)	↑ Mean power ↑ Mean anaerobic capacity ↑ Mean anaerobic power ↑ Mean peak power ↑Mean total work ↔ Mean fatigue index	N/A
2019	Lin *et al*.^(37)^	12 (M)	24·7	0·5	72·1	2·4	NR		Active	Randomised, double-blind, placebo-controlled, crossover trial	Chronic	8	400	NR	Treadmill (30-min run at 75 % VO2max)	NR	↓ CK (72 h post-exercise only) ↔ Lactate ↔ IL-1B ↔ IL-6 ↔ TNF-α ↔ CRP
2021	Williams *et al*.^(38)^	10 (M)	24·8	5·6	83·2	7·6	NR		Resistance-trained (8·7 ± 6·3 years experience)	Randomised, double-blind, placebo-controlled, crossover trial	Acute	4	1500 for 3 d 500 additional 30-min pre-exercise	1 % 3 %	Resistance exercise (bench press)	↑ Mean concentric velocity ↓ Total repetitions (RTF)	↑ Lactate ↔ Epinephrine ↑ Norepinephrine
2023	Liu *et al*.^(39)^	48 (M)	20·5	2·6	75·1	7·0	24·4	1·7	Untrained	Randomised, double-blind, placebo-controlled, crossover trial	Chronic	30	2400	0·5 % NR	Resistance exercise (squat and bench press)	↑ 1RM (bench press) ↑ 1RM (squat) ↑ MVIC (knee extension) ↑ RTF (bench press)	N/A

1-RM, 1-repetition maximum; ALT, alanine transaminase; AST, aspartate aminotransferase; BM, body mass; CK, creatine kinase; CRP, C-reactive protein; DOMS, delayed onset muscle soreness; EE, energy expenditure; eHSP72, extracellular heat-shock protein 72; F, female; G-CSF, granulocyte-colony stimulating factor; GH, growth hormone; GPx, glutathione peroxidase; HR, heart rate; M, male; MCP-1, monocyte chemoattractant protein-1; MVIC, maximum voluntary isometric contraction; *n,* number of participants; N/A, not applicable; NR, not reported; PCr, phosphocreatine; Pi, inorganic phosphate; PL, placebo; RPE, ratings of perceived exertion; RR, *Rhodiola rosea*; RTF, repetitions to failure; SOD, superoxide dismutase; TAC, total antioxidant capacity; TBARS, thiobarbituric acid reactive substances; TTE, time-to-exhaustion.

* Acute is defined here as 1–7 d of supplementation, with chronic defined as >7 d of supplementation.

#### Exercise protocols

Eight of the clinical studies identified included bicycle ergometry as an exercise testing modality, and the specific protocols varied widely^(23,24,25,28,29,30,35,36)^. Three trials included running, either on a treadmill^(33,37)^ or as a marathon race^(31,32)^, one trial employed walking on a treadmill in a climate chamber^(34)^, one trial used rowing ergometry^(27)^ and three trials incorporated resistance exercise, including wrist flexion^(26)^, bench press^(38,39)^ and/or squat exercises^(39)^.

#### Participant demographics

The participants in most trials were healthy young adults, with mean ages <30 years in all trials except one, which contained participants with mean ages of ∼40 to 44 years and was described in two articles^(31,32)^. Eleven studies included only male participants, two only included female participants, two included both sexes and biological sex was not reported in one investigation. Training status ranged from untrained to highly trained, although some descriptions of training status were vague, precluding the ability to determine the true training status of participants. Nonetheless, training statuses were designated by study authors as untrained (*n* 2), recreationally active (*n* 4), active (*n* 4) and trained/athletes (*n* 5), with training status not reported in one investigation. Collectively, based on a recent Participation Classification Framework^(40)^, most participants likely fell within the tier 1 (recreationally active) or tier 2 (trained/developmental) categories, with the possibility of some untrained participants falling within tier 0 (sedentary)^(24,39)^ and some highly trained participants belonging to tier 3 (highly trained/national level)^(27)^. Six studies used acute *RR* supplementation protocols^(26,29,30,33,36,38)^, defined here as 1–7 d of supplementation, nine implemented chronic supplementation (>7 d; range 8–38 d)^(23,24,27,28,31,32,34,37,39)^ and one incorporated both acute and chronic supplementation strategies^(25)^. Daily doses of *RR* ranged from 100 to 2400 mg/d. While not all studies reported the concentration of bioactive compounds, the most commonly reported concentrations were ∼1 % salidroside and ∼3 % rosavin.

### Endurance exercise capacity and performance

Several trials included in this review assessed the effects of *RR* on endurance exercise performance. One crossover study of ‘physically active’ males and females reported 2·4 % longer cycling TTE following acute *RR* ingestion^(25)^, and a separate parallel-arm trial of male physical education students ‘not engaged in high-performance sports’ at the time of testing found a similar increase in cycling TTE of 2·6 %, although this was not statistically significant^(35)^. Both studies provided a 200 mg dose of *RR* (3 % rosavin) 60–90 min before the incremental maximum effort TTE test. However, one trial evaluated TTE both after acute (2-d) and chronic (28-d) supplementation^(25)^, whereas the other only tested TTE after chronic (28-d) supplementation^(35)^. In the study of De Bock *et al*.^(25)^, an increase in TTE (+24 s, on average; *RR* 17·2 (se 0·8) min *v*. placebo 16·8 (se 0·7) min) was only observed in the acute crossover trial, and there were no between-condition differences in the subsequent parallel-arm trial included in the same report, which included 200 mg/d *RR* supplementation over 4 weeks. This could be due to the larger sample size per condition/group and greater statistical power in the acute trial (*n* 24, crossover design) compared with the chronic trial (*n* 11–12 per group, parallel arm). Similarly, the trial of Jówko *et al*.^(35)^ used chronic supplementation (600 mg/d, with 200 mg/d provided before TTE tests) in a parallel-arm design (*n* 13 per group) and found no statistically significant effects of chronic supplementation on TTE. However, the mean difference in TTE in the *RR* group was +20·8 s (+2·6 %) after 4 weeks of supplementation compared with −10·1 s (-1·3 %) with placebo. As such, it is possible these trials of chronic supplementation were underpowered to detect a small-but-meaningful influence of supplementation on TTE performance. Additionally, analysis of changes in maximal cycling power from the incremental maximum effort TTE tests indicated a significant difference between *RR* (+5·7 %) and placebo (-4·1 %)^(35)^.

Other studies have yielded conflicting or null results. In a crossover study including recreationally active females, *RR* improved time trial performance in a 6-mile bicycle ergometry test (*RR* 25·4 (se 2·7) min, placebo 25·8 (se 3·0) min, *P* = 0·04) following acute supplementation with 3 mg/kg (∼170 mg) *RR* provided 60 min before exercise^(29)^. *RR* also reduced average heart rate during the warm-up period (*RR* 136 (se 17) bpm, placebo 140 (se 17) bpm). In contrast, supplementation did not affect 2000-m rowing time in male rowers^(27)^ or marathon performance in male and female runners^(31,32)^. Additional research reported no benefit of acute *RR* supplementation for 5-km run time trial performance in recreationally active males^(33)^, nor any benefit of chronic supplementation for a treadmill walk to exhaustion conducted in a climate chamber^(34)^. However, a separate trial found *RR* decreased heart rate during bicycle ergometry work capacity testing following 20 d of supplementation with 100 mg/d^(23)^. These findings are discordant with other investigations reporting no influence of *RR* on heart rate during exercise^(28,30,33,34,35)^. The divergent training statuses, exercise testing modalities and *RR* dosing protocols may contribute to differences in endurance exercise performance outcomes.

As discussed, improvements in TTE with *RR* supplementation in murine models have been associated with higher resting liver glycogen content and attenuated exercise-induced reductions in muscle glycogen^(12)^, suggesting that alterations in glycogen turnover potentially contribute to ergogenic effects on endurance exercise following chronic supplementation. *RR* has also been found to support mitochondrial ATP content^(13)^, representing another mechanism by which *RR* may improve prolonged exercise performance. However, similar outcomes have not been examined in human participants to determine whether these mechanisms contribute to the observed results.

### Power and resistance exercise performance

Although limited, clinical research examining the effects of *RR* supplementation on power and resistance exercise performance has demonstrated potentially meaningful ergogenic effects. In their study of physically active (>150 min/week of moderate physical activity) young adult females, Ballmann *et al*.^(36)^ observed improvements in nearly all outcomes during repeated Wingate tests (3 × 15-s tests) performed on a bicycle ergometer, including mean and peak power, total work, anaerobic capacity and anaerobic power following *RR* supplementation. Effect sizes indicating the magnitude of performance improvements ranged from small to large, with the largest values observed for anaerobic capacity (*RR* 10·5 (se 0·9) watts/kg body mass, placebo 10·1 (se 1·1) watts/kg; *P* = 0·01; Cohen’s d effect size 0·96 (large)) and anaerobic power (*RR* 15·2 (se 1·1) watts/kg body mass, placebo 14·0 (se 1·2) watts/kg; *P* = 0·03; Cohen’s d effect size 1·07 (large)). The supplementation protocol included 3 d of 1500 mg/d *RR* (1 % salidroside, 3 % rosavin), followed by 500 mg ingestion on the fourth day, 30 min prior to exercise testing. This trial, along with a subsequent trial from the same research group^(38)^, used a higher dose of *RR* than nearly all other trials we identified (Table 1). The origin of this higher dose appears to be a study conducted by Walker *et al*.^(26)^, who sought to employ a dose higher than manufacturer recommendations in an attempt to approach doses shown to exert beneficial effects on mitochondrial ATP content in rodents^(13)^. While one other investigation used a higher total dose of *RR* (2400 mg/d), the lower concentration of bioactive compounds (0·5 % salidroside, rosavin not reported)^(39)^ led to a lower absolute dose of these compounds compared with the aforementioned studies. The trial of Walker *et al*.^(26)^ first administered a daily dose of 1500 mg/d (3 % rosavin, salidroside not reported), although this was in the context of a muscular endurance test (incremental forearm wrist flexion to exhaustion). In this instance, there were no benefits of acute (4-d) supplementation, although the forearm exercise protocol is dissimilar to those employing multiple larger muscle groups and inducing greater systemic stress and fatigue. As discussed, other research also supports potential benefits of *RR* for improving maximal cycling power^(35)^.

Two recent trials have examined whether *RR* supplementation improves resistance exercise performance, with one employing acute supplementation prior to exercise testing^(38)^ and the other pairing chronic supplementation with supervised resistance training^(39)^. In untrained participants, Liu *et al*.^(39)^ found that 30 d of supplementation with 2400 mg/d *RR* (0·5 % salidroside, rosavin not reported) alongside supervised resistance training produced superior performance adaptations compared with placebo. The resistance training programme contained thirteen training sessions over the 30-d study and included the bench press and deep squat exercises, with 4 sets of 10 repetitions at 60 % of the pre-training 1-repetition maximum (1RM) for the first 15 d and 4 sets of 8 repetitions at 70 % of the pre-training 1RM for the last 15 d. In the *RR* group, greater increases were observed for bench press 1RM (9 % greater increase with *RR* compared with placebo; *P* = 0·01), squat 1RM (7·5 % greater increase with *RR* compared with placebo; *P* = 0·01), knee extension maximal voluntary isometric contraction (8·6 % greater increase with *RR* compared with placebo; *P* = 0·008) and bench press repetitions to failure (12·7 % greater increase with *RR* than placebo; *P* = 0·005). The same study also found potentially additive effects of *RR* and caffeine ingestion in untrained participants, leading to a follow-up examination of *RR* plus caffeine in resistance-trained participants. This subsequent work also found improvements in select resistance exercise performance metrics compared with placebo^(39)^. Interestingly, Williams *et al*.^(38)^ observed some beneficial and some disadvantageous effects of acute *RR* supplementation (1500 mg/d, 1 % salidroside, 3 % rosavin) on bench press performance in resistance-trained males (8·7 (se 6·3) years resistance training experience). *RR* supplementation led to an ∼8 % greater increase in mean concentric velocity (*P* = 0·049; Cohen’s d effect size 0·73 (medium-to-large)) during a set of 2 repetitions at 75 % of 1RM, performed with maximal explosive intent, compared with placebo. However, in a subsequent test of repetitions to failure across 3 sets at 75 % 1RM, *RR* supplementation reduced total repetitions completed compared with placebo (*P* < 0·001; Cohen’s d 1·90 (large)), although the difference was small (∼2 repetitions across 3 sets). Although the test of concentric velocity can be viewed as non-fatiguing, and a 5-min rest took place between the velocity test and subsequent repetitions to failure protocol, it is possible the superior performance in the *RR* condition during the concentric velocity test influenced subsequent performance during the repetitions to failure test. Nonetheless, a potential tradeoff between mean concentric velocity and total training volume should be considered, as the relative importance of these variables depends on training goals and other contextual factors.

### Muscle damage and inflammation

Several trials have examined the potential influence of *RR* on post-exercise markers of muscle damage. While *RR* supplementation has been found to reduce creatine kinase concentrations at rest and following fatiguing bicycle ergometry tests^(24,28)^ and treadmill running^(37)^, this has not been found consistently in all studies^(27,31,32,35)^. When untrained adults performed an incremental bicycle ergometry test to exhaustion, Abidov *et al.* ^(24)^ found a substantial increase in creatine kinase concentrations (∼166 U/ml at baseline to ∼1650 U/ml 5 h after exercise), which did not appear to be influenced by *RR* supplementation (30 d of 680 mg/d prior to test). However, 5 d after the test, creatine kinase fell to ∼1450 U/ml in *RR* condition, whereas values remained at ∼2750 U/ml in the placebo condition, on average, suggesting a delayed effect of *RR*. In the same study, *RR* supplementation reduced C-reactive protein both 5 h and 5 d after the exercise test. Following 30 d of supplementation with 170 mg *RR*/d, Parisi *et al*.^(28)^ reported lower creatine kinase concentrations compared with placebo, both at rest and during exercise recovery. Most of the investigations showing no effect of *RR* supplementation on creatine kinase and other markers of muscle damage or inflammation have included well-trained participants, such as marathon runners^(31,32)^ and members of a national rowing team^(27)^. As such, it is plausible that adaptations to habitual exercise training – such as the neural, connective tissue and cellular factors potentially contributing to the repeated bout effect^(41)^ – either minimised muscle damage or otherwise reduced the likelihood of an influence of supplementation. Additional trials have reported no changes in multiple markers of inflammation, including C-reactive protein, various IL and liver enzymes following treadmill or marathon running^(31,32,37)^.

### Energy systems

As mentioned, *RR* influences ATP production, energy substrate storage and signalling pathways involved in cellular energy status in rodents^(12,13,16)^. A few clinical studies have investigated outcomes related to energy metabolism. For instance, *RR* supplementation has been found to increase^(38)^, decrease^(28)^ or exert no discernible influence^(27,29,33,34,37)^ on post-exercise lactate concentrations compared with placebo. The sole study reporting an increase in lactate concentrations with supplementation used resistance training in resistance-trained males^(38)^, while the only trial indicating a decrease in post-exercise lactate included cycling to exhaustion in male athletes^(28)^. The studies reporting no influence on lactate used varying exercise modalities and participants (i.e. rowing in male rowers^(27)^, treadmill running in active males^(33,37)^, cycling in recreationally active females^(29)^ and treadmill walking in active males^(34)^).

As mentioned, *RR* supplementation has been reported to improve Wingate test performance, implying potential benefits related to the energy substrates and metabolic pathways involved in rapid ATP production (i.e. ATP storage, the ATP-phosphocreatine system and anaerobic glycolysis). However, one of the most informative studies relevant to these outcomes^(26)^ demonstrated no clear influence of *RR* on the phosphocreatine energy system. Specifically, there were no differences in concentrations of phosphocreatine, ATP, inorganic phosphate or pH during incremental wrist flexion performed to exhaustion in resistance-trained (> 6 months training experience) males following 4 d of *RR* supplementation (1500 mg/d for 3 d, 1000 mg on the testing day; 3 % rosavin). The authors speculated the lack of effect on muscle phosphate kinetics, in contrast to rodent research^(13)^, could have been related to supplement dosing. Specifically, they estimated that a dose of ∼4000 mg/d *RR* for humans would be needed to match the dose provided by Abidov *et al*.^(13)^, who reported superior mitochondrial ATP preservation following exercise in rodents supplemented with *RR.* Additionally, the authors acknowledged that if *RR* influences central fatigue, the model of wrist flexion may not be optimal to detect ergogenic effects^(26)^. Other relevant effects observed in murine models, such as increased glycogen storage and delayed glycogen depletion^(12)^, have yet to be examined in humans.

### Antioxidant activity

Multiple bioactive compounds within *RR* have antioxidant activity in rodent models^(19,20)^. In human trials, two studies have reported an increase in total antioxidant capacity, an overall measure of plasma antioxidant activity, following 28 d of 200–600 mg/d *RR* supplementation in recreationally active or highly trained individuals^(27,35)^. This was observed for resting (pre-exercise) values in both studies and for post-exercise values in one trial^(27)^. The differences in exercise testing protocol (i.e. 2000-m maximal effort rowing^(27)^ *v*. incremental maximal effort bicycle ergometry test^(35)^) and participant training status (national rowing team members^(27)^ *v*. recreationally active^(35)^) may have influenced the difference in post-exercise total antioxidant capacity values. Alongside the increase in total antioxidant capacity, a decrease in post-exercise erythrocyte superoxide dismutase activity was observed by Skarpanska-Stejnborn *et al*.^(27)^, which the authors interpreted as an indication that *RR* led to more effective elimination of superoxide anions in erythrocytes. However, Jówko *et al*.^(35)^, who also reported increased total antioxidant capacity at rest, found no effect of supplementation on erythrocyte superoxide dismutase activity at rest, post-exercise or 24 h after exercise. Additional research is needed to clarify the degree to which *RR* exerts antioxidant effects in humans. Based on the lack of investigations reporting ergolytic effects of supplementation, coupled with several trials reporting positive effects, the current evidence does not suggest that any supplementation-associated increases in antioxidant status compromise exercise performance in the short term^(18)^. However, while ten studies employed *RR* supplementation lasting 8–38 d, most investigations have not included supervised or structured exercise training alongside supplementation. As such, the influence of long-term *RR* supplementation on *adaptations* to chronic exercise training is currently unclear. Future research should employ adequately powered parallel-arm trials to establish whether long-term *RR* supplementation alongside progressive exercise training influences adaptations to exercise, not only in terms of antioxidant capacity but also for exercise performance, body composition and other exercise- and health-relevant outcomes.

### Other outcomes

In addition to the effects of *RR* on exercise performance, muscle damage, inflammation, energy systems and antioxidant activity, a few other outcomes from trials in humans are noteworthy. For instance, some research groups have documented reductions in ratings of perceived exertion following acute *RR* supplementation (3 mg/kg; ∼170 to 203 mg) provided 30 min prior to time trial cycling^(29)^ or cycling with a fixed duration and intensity^(30)^. In one of these trials, reduced ratings of perceived exertion were concurrent with increases in self-reported arousal, pleasure and vigour^(30)^. These beneficial subjective responses are aligned with other documented effects of *RR*, such as its anti-stress and anti-fatigue effects^(8,9)^. While speculative, these effects could possibly be due to the influence of bioactive compounds on monoamine neurotransmitters and opioid peptides^(10)^. However, several other trials have reported no influence of supplementation on ratings of perceived exertion^(26,28,33,34)^. Due to potential antidepressant properties of *RR* ^(42)^, the possibility of varying effects, including downstream consequences for exercise performance, based on the presence of affective disorders should be considered. Furthermore, it should be noted that *RR* has been widely studied for aiding mental health and cognitive function^(11)^ and promoting resistance to general stress and fatigue^(10)^, although the purpose of the present review is to describe the roles of *RR* within the context of physical performance.

Additional trials have reported beneficial effects of *RR* supplementation that may be relevant to sports performance, such as improvements in movement speed and accuracy, reductions in mental fatigue^(23)^ and quicker reaction time^(35)^. Spasov *et al*.^(23)^ found that 20 d of supplementation with 100 mg/d *RR* (2 % salidroside, 3 % rosavin) reduced self-assessed mental fatigue, with a ∼30 % decrease in fatigue – based on a questionnaire evaluating various forms of fatigue, sleeping patterns, mood and mental states – compared with a ∼21 % increase in the placebo group. In a study of 28 d of supplementation with 600 mg/d *RR* (3 % rosavin), Jówko *et al*.^(35)^ found significant differences in relative changes in simple reaction (median reaction time and total response time) and choice reaction (number of correct responses) with supplementation compared with placebo. Median reaction time and total response time decreased by 5·7–9·5 % on average with supplementation, with mean increases of ∼4 to 5 % in the placebo group. The increase in correct responses in the choice reaction test was 16 % on average with supplementation compared with an increase in 6·6 % on average with placebo. However, changes in other reaction metrics, such as median movement time during the simple reaction test and median response time in the choice reaction test, were unaffected by supplementation^(35)^. Collectively, these findings provide initial support for benefits of *RR* for cognitive and subjective outcomes ancillary to exercise performance per se.

While limited data are available, one trial assessed the potential immunomodulatory actions of *RR* in exercising adults. After 30 d of supplementation with 600 mg/d *RR* or placebo, male and female runners provided serum samples before and after completing a marathon race, to examine various components of the immune system^(32)^. While *RR* did not exert any obvious antibacterial effects, a decrease in viral replication following vesicular stomatitis virus was evident in the serum of runners supplementing with *RR*, leading the authors to conclude that supplementation could help defend against exercise-induced susceptibility to viral infections^(32)^. If this is the case, the finding is potentially meaningful due to the detrimental impacts of acute illnesses in athletes, the inability to train and compete due to infection and the risk of infection transmission to team members^(43)^.

## Recommendations for supplementation

### Supplement quality and third-party testing

As with any dietary supplement, practitioners and consumers considering supplementation with *RR* should ensure a high-quality product from a reputable manufacturer is selected. Currently, third-party testing is one of the most objective ways to ensure the purity, quality and ingredient doses within a product. Examples of well-recognised third-party testing organisations include NSF, Informed, U.S. Pharmacopeia, the Banned Substances Control Group and Labdoor. Those who may be subject to drug testing, particularly athletes, may benefit from the rigour of testing associate with services such as NSF Certified for Sport® and Informed Sport. While details of specific testing services vary, most include components of verification of the stated ingredients and doses, examination to ensure banned or dangerous substances are not present and confirmation of good manufacturing practices. Further details of each testing service are available on their respective websites.

### Salidroside and rosavin concentration

While commercially available *RR* preparations vary in their concentration of the primary active components, it is recommended to choose a product that states the concentration of salidroside and rosavin within the product, preferably with verification of these concentrations via a certificate of analysis or third-party testing. As described, many of the *RR* preparations discussed in the present review contained ∼1 % salidroside and ∼3 % rosavin, although variability was present (Table 1). As described in the following sections, most studies demonstrating ergogenic effects for either endurance or strength and power outcomes have also used this common concentration of salidroside and rosavin.

### Supplementation for endurance outcomes

The disparity in *RR* doses and mixed findings in extant research preclude definitive recommendations for an optimal dosing protocol. However, the trials reporting ergogenic effects of supplementation can be examined to provide tentative recommendations. Ergogenic effects of *RR* for TTE during an incremental bicycle ergometer test lasting ∼17 min have been seen at doses of 200 mg (1 % salidroside, 3 % rosavin)^(25)^, with a benefit for time to completion during a 6-mile bicycle ergometer time trial lasting ∼25 min observed at ∼170 mg (3 mg/kg; 1 % salidroside, 3 % rosavin)^(29)^. These studies were conducted in ‘active’ or ‘recreationally active’ individuals. While other trials have shown no such benefits at similar doses, a tentative recommendation of an absolute dose of ≥200 mg *RR* containing ≥1 % salidroside and ≥3 % rosavin, consumed 60 min prior to exercise, may be appropriate for those planning to consume *RR* for the purpose of enhancing endurance exercise performance or capacity. These doses are also consistent with those producing some of previously discussed effects, albeit inconsistently, of reducing heart rate, improving subjective outcomes (e.g. general well-being, mental fatigue, vigour) and increasing total antioxidant capacity. To date, the highest located doses of *RR* employed the context of endurance exercise capacity and performance are 600–680 mg^(24,31,35)^, with primarily null results for exercise performance, except for changes in maximum power during a bicycle ergometry maximum effort test^(35)^. However, these doses are less than half the 1500–2400-mg doses employed in models of forearm wrist flexion^(26)^, Wingate anaerobic testing^(36)^ and resistance exercise^(38,39)^.

### Supplementation for strength and power outcomes

When compared with endurance exercise trials, the studies reporting ergogenic effects of *RR* for strength and power outcomes have generally utilised higher doses. Two trials found benefits of 1500 mg/d *RR* (1 % salidroside, 3 % rosavin) on various metrics of power during repeated 15-s Wingate anaerobic tests on a bicycle ergometer and mean concentric velocity during the bench press exercise. An additional study found a benefit of 2400 mg/d *RR* (0·5 % salidroside) on adaptations to 30 d of resistance training, although this was in untrained individuals. Based on the total dose and concentration of salidroside, these trials collectively used ∼12 to 15 mg salidroside. Using the common concentrations of ∼1 % salidroside and ∼3 % rosavin, a potentially ergogenic dose of *RR* for strength and power outcomes based on current research would be ∼1500 mg/d, with ∼500 mg consumed within 1 h before commencing exercise. Importantly, the apparent difference in recommended *RR* dose for endurance *v*. strength/power outcomes is based on the doses investigated in existing research rather than a comprehensive dose–response examination to inform whether ergogenic doses truly vary between these categories. As such, future research is needed to clarify and confirm the optimal doses of *RR* for those seeking to improve diverse exercise performance outcomes.

### Training status

The current literature does not allow for definitive conclusions regarding the effects of exercise training status on *RR* supplementation recommendations. Most studies (13/16) were conducted in participants who were ‘recreationally active,’ ‘active’ or ‘trained,’ sometimes referred to as ‘athletes.’ In most cases, there was limited information regarding the precise meaning of these descriptors and terms were used disparately across studies. Collectively, as most studies likely contained participants in the tier 1 (recreationally active) or tier 2 (trained/developmental) categories^(40)^, the current literature is generally not conducive to establishing whether *RR* supplementation differentially affects individuals of differing training statuses.

### Sex differences

While potential sex differences related to *RR* supplementation are worth considering, the current literature has limited ability to inform this discussion. As noted, eleven studies included only male participants, two only included female participants, two included both sexes and biological sex was not reported in one investigation (Table 1). Additionally, the two investigations including both males and females did not present detailed sex-specific analysis^(25,31)^. Future research that is adequately powered to examine sex differences will help provide guidance regarding the possibility of sex-specific effects of supplementation.

## Summary and future directions

Regarding exercise performance, *RR* supplementation has demonstrated select benefits for improved TTE during incremental maximum effort testing and time trial performance, both using bicycle ergometry. However, null results have also been observed in several trials using running, walking and rowing exercise. Adequately powered trials incorporating different doses of *RR* are needed to establish the effects of supplementation more clearly, and meta-analysis of exercise-relevant outcomes may provide additional insight. Interestingly, several trials have reported reduced heart rate or ratings of perceived exertion during exercise with *RR* supplementation, which could relate to the adaptogenic properties of the compounds within this plant. However, other trials have failed to confirm this effect. While less studied than endurance exercise, initial studies have indicated the potential for ergogenic effects of *RR* supplementation on power during anaerobic capacity and resistance exercise tests. Importantly, these trials have also administered higher doses of *RR* (1500–2400 mg/d; 0·5–1 % salidroside, ∼3 % rosavin) compared with much of the work on endurance exercise (100–680 mg/d; typically ∼1 % salidroside and ∼3 % rosavin, when reported). As such, dissimilarity in doses of bioactive components of *RR* should be considered, in addition to the potential for *RR* to differentially influence disparate types of physical performance. The optimal doses of different *RR* bioactives to maximise performance benefits in different exercise contexts have not yet been established. Future trials examining multiple dosing protocols using standardised *RR* extracts with known concentrations of salidroside and rosavin may illuminate optimal dosing protocols as well as whether acute and chronic supplementation differentially affect physical performance. In the existing literature, both acute and chronic supplementation have produced, or failed to produce, ergogenic effects. While select ergogenic benefits have been observed for endurance exercise outcomes at relatively low doses of ∼200 mg *RR*, investigation of higher doses may be warranted due to the positive effects observed for doses of ≥1500 mg for strength and power outcomes. Conversely, whether the higher doses of *RR* for strength and power outcomes are truly needed is unclear due to the lack of research employing lower doses. As such, studies examining both endurance- and strength- or power-related outcomes would benefit from incorporating multiple doses of *RR* to better establish effective dosing. Additionally, based on the limited inclusion of female participants in extant research, future studies should better establish the effects of *RR* supplementation in females and explore whether sex differences in the efficacy of supplementation are observed. Finally, the current research minimally addresses the question of whether *RR* supplementation influences *adaptations* to chronic exercise training. As such, randomised, placebo-controlled, parallel-arm trials incorporating supervised exercise training should be conducted to help address this research gap in groups of varying training status.

Beyond exercise performance itself, *RR* may influence exercise-relevant outcomes, such as muscle damage, inflammation, substrate metabolism, antioxidant capacity and perceptions of activity. While some trials reported benefits of supplementation on markers of muscle damage and inflammation, others have failed to observe such effects, particularly in exercise-trained individuals. Most trials have not reported an influence of supplementation on lactate production, with some exceptions. During wrist flexion exercise, there was no clear influence of acute *RR* supplementation on the phosphocreatine energy system. Other components of energy storage and turnover have yet to be examined in humans, despite the beneficial effects of supplementation observed in murine models. Total antioxidant capacity, measured at rest or following exercise, may increase in humans following supplementation, although the influence on specific antioxidant enzymes has been inconsistent. Nonetheless, the current evidence does not support an ergolytic effect of *RR* due to its potential antioxidant activity. Some studies reporting subjective outcomes have demonstrated the potential for supplementation to reduce perceived exertion and improve other psychological variables, while others have reported no benefits. Finally, the limited research to date has supported select immunomodulatory effects of supplementation in exercising humans, but additional studies are needed to establish any clinical significance of these findings. Additional confirmatory studies are needed for the aforementioned outcomes, particularly using chronic supplementation of varying doses, due to the limited existing research. Within these examinations, elucidation of the mechanisms by which bioactive components of *RR* exert their physiological effects is also warranted. Importantly, while the focus of the present review was physical performance and related outcomes, it is noteworthy that *RR* has been investigated for numerous other performance-enhancing effects, notably those related to cognitive function and general well-being^(10,11,44,45,46)^.

While a comprehensive discussion *RR*’s safety profile is beyond the scope of the present review, this botanical was deemed to have ‘acceptable safety data’ by the World Federation of Societies of Biological Psychiatry and Canadian Network for Mood and Anxiety Treatments Taskforce^(47)^. Human data indicate *RR* and other *Rhodiola* species are well tolerated and produce minimal side effects^(2,48)^. Toxicological investigations in mice also indicate a low risk of toxicity^(2,49)^. Nonetheless, the potential for interactions between *RR* and pharmaceuticals or dietary supplements should be explored in future research. Additionally, while not discussed here, other *Rhodiola* species, such as *Rhodiola crenulata*, may warrant investigation for potential ergogenic effects^(13,50)^. Importantly, in addition to some trials not reporting the concentration of bioactive compounds within *RR*, most investigations do not include analytical confirmation of the purity of *RR*, even when concentrations of bioactive compounds are stated. As such, variation in the purity and true concentrations of bioactive components in *RR* supplements used in extant research could help explain divergent effects.

To conclude, *RR* has the potential to enhance performance and performance-related outcomes in several types of exercise; however, the current literature does not unanimously show ergogenic effects of supplementation with this plant. Variability in the supplementation dose and duration, concentrations of bioactive compounds, participant characteristics, exercise tests employed and statistical power may in part explain the disparate findings in the existing literature. While studies to date provide informative first steps, subsequent investigation of the potential ergogenic effects of *RR* will undoubtedly help clarify remaining questions regarding effects of *RR* based on supplementation protocol, exercise modality, exercise training status and more. The longstanding use of *RR,* the existing animal research and contemporary clinical trials in humans indicate that this botanical may have physiological effects that enhance exercise performance and related outcomes.
